# Anti-diabetic combination therapy with pioglitazone or glimepiride added to metformin on the AGE-RAGE axis: a randomized prospective study

**DOI:** 10.3389/fendo.2023.1163554

**Published:** 2023-08-11

**Authors:** Eugenio Ragazzi, Silvia Burlina, Chiara Cosma, Nino Cristiano Chilelli, Annunziata Lapolla, Giovanni Sartore

**Affiliations:** ^1^ Department of Pharmaceutical and Pharmacological Sciences, University of Padova, Padova, Italy; ^2^ Department of Medicine – DIMED, University of Padova, Padova, Italy

**Keywords:** advanced glycation end products, s-RAGE, AGEs, type 2 diabetes mellitus, pioglitazone, glimepiride

## Abstract

**Introduction:**

The ratio between advanced glycation end products (AGEs) and soluble form of receptor (s-RAGE) has been proposed as a risk marker for renal and cardiovascular diseases. The aim of this study was to evaluate in the diabetes condition the influence of two different oral anti-diabetic treatments on the AGE/s-RAGE ratio, during a 5-year observation period.

**Methods:**

Seventy-three patients with type 2 diabetes mellitus were randomly assigned to a drug therapy with pioglitazone or glimepiride, combined to metformin. Each subject was evaluated at baseline and after 5 years of treatment.

**Results:**

In both groups s-RAGE levels did not significantly vary, while the levels of AGE and AGE/s-RAGE were both significantly reduced, basal compared to 5-year values. Within pioglitazone group, as well within glimepiride group, significant variations (Δ, as difference between 5 years of treatment minus basal) were observed for AGE (Δ= ˗21.1±13.4 µg/ml, *P*<0.001 for pioglitazone; Δ= ˗14.4±11.4 µg/ml, *P*<0.001 for glimepiride) and in AGE/s-RAGE (Δ= -0.037±0.022 µg/pg, *P*<0.001 for pioglitazone; Δ= -0.024±0.020µg/pg, *P*<0.001 for glimepiride), suggesting an average decrease of the parameters by more than 50% in both treatments. Pioglitazone was more effective than glimepiride in reducing AGE/s-RAGE ratio after 5 years of therapy.

**Conclusion:**

These data can help to explain the benefits of oral anti-diabetic therapy in relation to the reduction of cardiovascular risk, as suggested by variations in AGE/s-RAGE ratio as biochemical marker of endothelial function; in particular, treatment with pioglitazone seems to offer greater long-term benefit on AGE-RAGE axis.

## 1 Introduction

Advanced glycation end products (AGEs) are derived from the non-enzymatic glycation of various molecules, primarily proteins, but also lipids, and amino groups present in nucleic acids (1). AGEs are glyco-oxidation products involved in the development of diabetes complications, such as diabetic neuropathy, retinopathy and nephropathy (2, 3); they can also predict cardiovascular disease in Type 2 Diabetes Mellitus (T2DM) patients (4, 5).

Serum AGE levels do not necessarily correlate with fasting plasma glucose or HbA1c levels, as observed by both Kilhovd et al. (6) and Lapolla et al. (7); this probably derives from the fact that AGE turnover is independent of glucose levels. Even a long period of good metabolic control is unable to normalize the levels of glyco-oxidation products, and this demonstrates that hyperglycaemia causes persistent oxidative stress, which is capable, by itself and independently of glucose concentrations, to induce and potentiate AGE formation in patients with diabetes (7).

AGEs exert their pathogenetic action by interacting with specific cellular receptors (RAGEs) that bind AGEs in a saturable manner. Two isoforms of RAGEs have been described (8, 9), consisting of cleaved RAGE (c-RAGE) originated by proteolysis, and endogenous secretory RAGE (es-RAGE), formed by splicing of RAGE mRNAS (10). c-RAGE and es-RAGE, collectively named soluble RAGE (s-RAGE), circulate in the blood, since they do not have transmembrane domains (9). The exact biological role of s-RAGE is only partially understood (5). Evidence supporting the role of both s-RAGE and es-RAGE as biomarkers or endogenous protective factors against RAGE-mediated pathogenesis is emerging (11). It has been hypothesized that circulating s-RAGE levels may inversely reflect RAGE activity (12).

Reduced levels of s-RAGE have been detected in patients with coronary heart disease compared to controls (13), in patients with peripheral arterial disease (14), in patients with stroke (15) and in patients with dementia of cardiovascular origin (16). Lower levels of s-RAGE are detected in T2DM patients compared to controls and the reduction also correlates with increased cardiovascular risk (17). s-RAGE are inversely related to metabolic syndrome parameters, including systemic blood pressure (18), BMI (18, 19), serum triglycerides, and insulin resistance (19). Conversely, in one study, no correlation was found between s-RAGE and peripheral or autonomic neuropathy, but increased levels of s-RAGE were found in patients with T2DM and chronic renal disease compared to controls (20); moreover, a direct correlation was found between nephropathy, decreased glomerular filtration and s-RAGE (20) and baseline levels of s-RAGE were found to be predictive of chronic kidney disease progression (4). Furthermore, Nin et al. (21) reported an inverse association between s-RAGE and renal function.

The values of AGE and s-RAGE, taken individually within pathologies connected to the AGE–RAGE axis, are not yet of univocal interpretation. To overcome this limit, some authors have proposed studies aimed at identifying more effective markers as a predictor of disease. The AGE/s-RAGE ratio, which was found higher in non-diabetic patients with NSTEMI (non ST-segment elevation myocardial infarction) than in the control group, has been proposed as a possible biomarker/predictor for a cardiovascular event, with a better sensitivity than the s-RAGE value alone in identifying patients at risk (22). Subsequent studies conducted by Prasad et al. (23, 24) have further developed this theory, comparing pathological conditions characterized by reduced serum/plasma levels of s-RAGE (NSTEMI, thoracic aortic aneurysm, hyperthyroidism, hypercholesterolemia) and pathological conditions characterized by increased serum/plasma levels of s-RAGE (end-stage renal disease, ESRD). Both under conditions of reduced s-RAGE and in the group of patients with increased s-RAGE, the AGE/s-RAGE ratio was higher in the disease cases when compared with the respective control subjects and correlated with a more advanced stage of sickness. From these assumptions, the unified parameter AGE/s-RAGE ratio was developed and proposed as the best risk factor in interpreting pathologies associated with the AGE–RAGE axis, regardless of the levels of s-RAGE (24). The relevance of AGE/s-RAGE ratio has been evaluated in various clinical contexts as a cardiovascular risk marker. In the study conducted by Kajikawa et al. (25) the AGE/s-RAGE ratio resulted as an independent predictor of flow-mediated vasodilation (FMD), while the serum level of AGEs alone or s-RAGE were not associated with FMD, suggesting the role of the ratio as biomarker for endothelial dysfunction.

The aim of the present study was to evaluate the impact of two oral hypoglycaemic regimens, pioglitazone or glimepiride in association with metformin, after 5 years of treatment on the trend of the parameters AGE, s-RAGE and the cardiovascular risk marker AGE/s-RAGE ratio, in a cohort of patients with T2DM. A second objective was to investigate whether these indicators are correlated with anthropometric parameters, glyco-metabolic control, inflammatory status, and the presence of complications.

## 2 Materials and methods

### 2.1 Study design and patient selection

The study was a randomized, open, parallel group intervention study conducted based on data collected as a participating center in the multicenter clinical trial TOSCA.IT (Thiazolidinediones Or Sulphonylureas and Cardiovascular Accidents. Intervention Trial; NCT00700856, https://www.clinicaltrials.gov/ct2/show/NCT00700856). The aim of the trial was to evaluate the different outcomes, in terms of incidence of fatal and non-fatal cardiovascular events, efficacy on glycaemic control and major cardiovascular risk factors, safety, tolerability and costs, in two groups of patients with T2DM and inadequately controlled with metformin alone, subjected to a different therapeutic regimen with the use of oral glucose-lowering agents (a sulfonylurea, or a thiazolidinedione) for a long term therapy (26).

The study protocol was approved by the Ethics Review Committee/Institutional Review Board of the Coordinating Center and by the Ethics Committees of each individual participating center. The study complies with the Declaration of Helsinki and the EU guidelines on Good Clinical Practice for conducting clinical trials of medicines. Informed consents were signed by each patient in the pre-randomization phase; to each participant was clearly explained the right to abandon the project at any time and for any reason, without any kind of consequence.

The inclusion criteria considered for the selection of patients in the study were (details in (26)): males and females, aged between 50 and 75 years, diagnosed with T2DM for at least 2 years; BMI between 20 kg/m² and 45 kg/m²; current hypoglycaemic therapy regimen consisting exclusively of metformin at a dosage of 2 g/day, for at least 3 months; HbA1c value between 53 mmol/mol (7%) and 75 mmol/mol (9%).

The exclusion criteria considered were: patients diagnosed with Type 1 Diabetes Mellitus; therapy with thiazolidinediones within 6 months prior to the start of the study; contraindications or documented intolerance to metformin, pioglitazone or glimepiride; documented presence of cardiovascular events (coronary or cerebrovascular) occurring in the 3 months preceding the start of the study; proliferative retinopathy; plasma creatinine values >1.5 mg/dL; presence of documented congestive heart failure (NYHA class 1 or higher); chronic use of corticosteroid drugs; presence of leg ulcers or gangrene; documented presence of liver cirrhosis or severe liver dysfunction (ALT values > 2.5 times the normal cut-off); pregnancy or breastfeeding; documented presence of oncological disease; substance abuse; presence of any other condition not previously mentioned that could interfere with adherence to therapy or cause serious harm to the patient.

Seventy-three patients with T2DM attending the U.O.C. of Diabetology and Dietetics of Ulss 6 Euganea, Padova (Italy) fulfilling the inclusion criteria were recruited and subsequently randomized into two arms of the study, characterized by different treatment regimens:

1) metformin 2 g/day, with the addition of pioglitazone (thiazolidinedione), at a dosage of 15 mg/day;
2) metformin 2 g/day, with the addition of glimepiride (sulfonylurea) at a dosage of 2 mg/day.

Clinical and laboratory parameters were measured at the randomization visit and at the follow-up visit after 5 years of treatment. For each patient, the following anthropometric, clinical and haemato-chemical parameters were investigated: age, duration of diabetes, height, weight, BMI, waist circumference, diastolic and systolic blood pressure, serum creatinine and eGFR, glycated haemoglobin (HbA1c), serum C-reactive protein (CRP), AGE, s-RAGE, and AGE/s-RAGE. Basal 10-year ASCVD (Atherosclerotic Cardiovascular Disease) risk was calculated according to https://tools.acc.org/ascvd-risk-estimator-plus/#!/calculate/estimate/.

### 2.2 Laboratory measurements

Glycated haemoglobin HbA1c was measured in blood samples taken at time 0 and after 5 years, by high performance liquid chromatography (HPLC, Menarini Akray ADAM A1c HA-8180v), according to the standards of the International Federation of Clinical Chemistry (FICC) (27). The instrument utilizes a reversed-phase cation exchange chromatography measurement principle, with a dual-wavelength colorimetry detection method (420nm/500nm LED-photodiode), equipped with a Column unit 80V maintained at 40°C. Elution is obtained in a five-step phosphate buffered gradient with increasing ionic strength using 3 specific buffers (80A, 80B and 80CV). Resolution is 0.1% HbA1c NGSP units, with measurement range 3-20%. The analysis is performed on approximatively 4 µL of whole blood.

AGEs were measured using an enzyme-linked immunosorbent assay (ELISA) according to Makita et al. method (28). Briefly, microtiter ELISA plates were coated with AGE-BSA conjugate (10 pg/ml, dissolved in PBS) and incubated for 2h at room temperature. Wells were washed and subsequently blocked by incubation with a solution of PBS containing 2% goat serum, 0.1% BSA and 1 mM NaN_3_. After addition of competing antigen (AGE-protein samples or AGE-BSA standard) and anti-AGE polyclonal antibody (final dilution 1/1000), incubation for 3 h at room temperature and final washing, wells were developed with an alkaline phosphatase-linked anti-rabbit IgG (p-nitrophenyl phosphate as colorimetric substrate). AGE protein adducts in samples was determined by comparison with AGE-BSA standard curve. The assay range is 19-1600 ng/ml.

s-RAGE were assayed by ELISA method (ELISA Kit, manufactured by Cusabio Biotech Co. Ltd.). Briefly, antibody specific for s-RAGE were pre-coated onto a microplate, where samples or standards were added, so any s-RAGE is bound by the immobilized antibody. Following removal of unbound conjugates, a biotin-conjugated antibody specific for s-RAGE was introduced into the wells, and after washing, avidin conjugated Horseradish Peroxidase (HRP) was added and developed on adequate substrate for reading absorbance at 450nm in a microplate reader. The linear range of the sRAGE measurements was from 78 to 5000 pg/mL, and the inter- and intra-assay coefficients of variation for sRAGE were <10% and 8.0%, respectively.

### 2.3 Statistical analysis

Data are expressed as mean and standard deviation (SD). The statistical analysis was performed using JMP® Version Pro 17 software for Windows (SAS Institute Inc, Cary, NC, USA). Rough data were analyzed for the presence of outliers by using Tietjen-Moore test and by robust PCA outliers’ method, which identifies outliers in the residuals of a robust decomposition of the data into a low-rank matrix and a sparse matrix of residuals. The comparison of variables at time 0 (basal) and after 5 years of treatment was obtained as after treatment minus before treatment mathematical difference (delta, Δ); it follows that a negative delta value indicates a reduction in the value of the variable under examination after treatment, while a positive delta indicates an increase.

The statistical significance of the difference of each continuous variable in each arm of the study was evaluated using the Student’s *t* test for paired data, after checking the normality of the distribution of values, considering both the kurtosis index and the bell skewness. Data from the two groups of patients were compared using Student’s *t* test for unpaired data. The presence of a correlation between available parameters was evaluated with linear regression, through Pearson’s *r* correlation coefficient. Pearson’s chi-squared (χ^2^) test was used to compare categorical variables. To detect overall distribution of some key parameters, the non-parametric technique of cluster analysis was used. Statistical significance for the differences was set at a *P* value < 0.05.

## 3 Results

Basal anamnestic and anthropometric parameters, as well as clinical, metabolic and inflammation parameters of the two groups of patients, treated with pioglitazone or glimepiride, are illustrated in **Table 1**. The two groups of patients present similar baseline characteristics, except for a significant difference in height (*P*=0.0183).

**Table 1:** Clinical and metabolic parameters of the two groups of patients.

Parameter	Pioglitazone (*n*=36)		Glimepiride (*n*=37)		*P^†^*	*P^†^*
	basal	5-year	basal	5-year	basal	5-year
Age (y)	63.5 ± 7.2	–	64.5 ± 6.4	–	0.5178	–
Male/Female (n)	24/12	–	15/22	–	0.3688	–
Smokers (yes/no)	4/32	–	6/31	–	0.5293	–
FPG (mg/dl)	132.2 ± 21.1	131.0 ± 19.6	127 ± 18.2	122.3 ± 19.5	0.6003	0.3327
Total cholesterol (mg/dl)	176.6 ± 31.9	170.6 ± 28.9	181.5 ± 37.4	178.1 ± 35.6	0.5487	0.3292
HDL cholesterol (mg/dl)	45.4 ± 10.0	49.9 ± 13.2	48.2 ± 13.8	47.0 ± 13.5	0.3197	0.3872
Triglycerides (mg/dl)	137.5 ± 52.3	120.0 ± 41.4	144.8 ± 71.4	137.5 ± 58.0	0.6149	0.1422
Antihypertensive drugs (yes/no)	25/11	–	26/11	–	0.9387	–
Lipid-lowering drugs (yes/no)	20/16	–	20/17	–	0.8974	–
Antiplatelet drugs (yes/no)	15/21	–	14/23	–	0.7382	–
10-year ASCVD risk (%)	20.4 ± 11.0	–	21.7 ± 13.9	–	0.6578	–
Diabetes duration (y)	7.14 ± 4.81	–	8.95 ± 5.10	–	0.1240	–
Weight (kg)	83.10 ± 14.62	85.87 ± 14.89**	80.47 ± 15.36	80.97 ± 15.82	0.4574	0.1775
Height (m)	1.69 ± 0.10	–	1.64 ± 0.10	–	*0.0183*	–
BMI (kg/m²)	28.89 ± 4.16	29.90 ± 4.52**	29.84 ± 4.37	30.05 ± 4.63	0.3443	0.8886
Waist circumference (cm)	101.11± 10.92	99.36 ± 19.17	99.97 ± 10.96	100.41 ± 10.74	0.6581	0.7740
SBP (mmHg)	132.03 ± 11.89	134.44 ± 9.40	135.38 ± 11.70	139.30± 10.63	0.2290	*0.0426*
DBP (mmHg)	83.64 ± 8.60	80.89 ± 6.60	80.86 ± 6.91	81.19 ± 6.62	0.1326	0.8468
HbA1c (%)	7.67 ± 0.37	7.43 ± 0.87	7.71 ± 0.42	7.18 ± 0.82**	0.6599	0.2056
eGFR (ml/min/1.73m²)	92.41 ± 9.60	84.38 ± 15.12	92.87 ± 9.57	85.91 ± 15.51	0.8374	0.6720
Serum creatinine (mg/dl)	0.80 ± 0.12	0.85 ± 0.17*	0.74 ± 0.17	0.78 ± 0.21	0.0903	0.1216
CRP (mg/dl)	0.23 ± 0.25	0.26 ± 0.45	0.42 ± 0.60	0.35 ± 0.35	0.0794	0.3387

^†^ *P*, comparison between pioglitazione vs glimepiride at different times. For continuous data, Student’s *t* test; for categorical data, chi-squared test.

**P<0.01, * *P*<0.05, basal vs 5-year for each respective patient group.

FPG, fasting plasma glucose; BMI, body mass index; SBP, systolic blood pressure; DBP, diastolic blood pressure; eGFR, estimated glomerular filtration rate; CPR, C-reactive protein.

10-year ASCVD risk was calculated according to https://tools.acc.org/ascvd-risk-estimator-plus/#!/calculate/estimate/.

After the 5-year treatment period, no significant differences were found between groups, except for a marginal significance of systolic blood pressure (*P*=0.0426). Within pioglitazone group, modest albeit significant variations were observed for weight (83.10 ± 14.62 *vs* 85.87 ± 14.89 kg, *P*<0.01), BMI (28.89 ± 4.16 *vs* 29.90 ± 4.52 kg/m², *P*<0.01), and serum creatinine (0.80 ± 0.12 *vs* 0.85 ± 0.17 mg/dl, *P*<0.05). Within glimepiride group, a difference after the 5-year treatment period was detected only for HbA1c which resulted significantly reduced (7.71 ± 0.42 *vs* 7.18 ± 0.82%, *P*<0.01).

**Figure 1:**
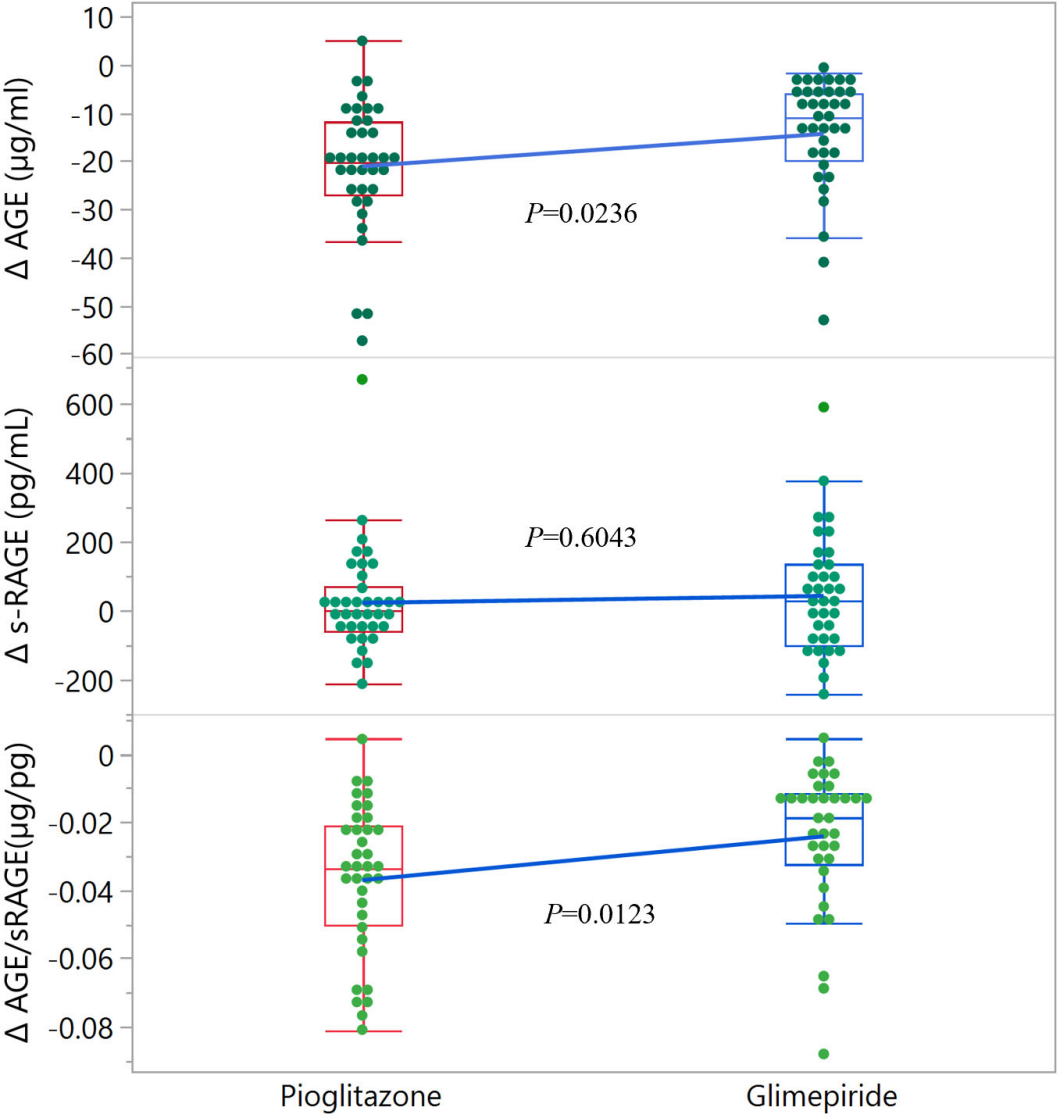
Distribution of Δ values for glyco-oxidation parameters in the two groups of patients. The edges of the boxes indicate the 25^th^ and 75^th^ quantiles, including the middle 50 percent of the data; whiskers indicate the furthest points within 1.5 x IQR from the box. IQR is the interquartile range, defined as the difference between the 75^th^ and 25^th^ percentiles. Statistical significance is indicated for each parameter.

With respect the specific parameters of glyco-oxidation (**Table 2**), basal levels of AGE, s-RAGE and the AGE/s-RAGE ratio were similar at basal time in the two treatment groups. After the 5-year treatment period, considering absolute values, no significant variations were found between groups for all the parameters; however, when considering mathematical differences (Δ, 5-year – basal), a significant difference between the two groups was found for Δ AGE level (*P*=0.0236), which then affected also the ΔAGE/s-RAGE ratio (*P*=0.0123) (**Table 2** and **Figure 1**). Within pioglitazone group, as well within glimepiride group, significant variations were observed for ΔAGE (Δ= -21.1 ± 13.4 µg/ml, *P*<0.001 for pioglitazone; Δ= -14.4 ± 11.4 µg/ml, *P*<0.001 for glimepiride) and in the ΔAGE/s-RAGE ratio (Δ= -0.037 ± 0.022 µg/pg, *P*<0.001 for pioglitazone; Δ= -0.024 ± 0.020µg/pg, *P*<0.001 for glimepiride) (**Table 2**), suggesting an average decrease of the parameters by more than 50% in both treatments.

**Table 2:** Comparisons for AGE, s-RAGE and AGE/s-RAGE data in the two groups of patients at both considered times.

	Parameter	basal	5-year	Δ 5-year - basal	Δ % 5-year - basal	*P^†^*
**Pioglitazone group** (*n*=36)	AGE (µg/ml)	28.9 ± 13.7	7.8 ± 6.9	-21.1 ± 13.4	-68.6 ± 32.4%	*<0.001*
	s-RAGE (pg/ml)	574.8 ± 93.8	595.9 ± 156.7	21.1 ± 150.3	4.7 ± 25.3%	0.4062
	AGE/s-RAGE (µg/pg)	0.051 ± 0.025	0.014 ± 0.014	-0.037 ± 0.022	-70.2 ± 27.1%	*<0.001*
**Glimepiride group** (n=37)	AGE (µg/ml)	24.6 ± 10.1	10.2 ± 6.7	-14.4 ± 11.4	-54.1 ± 26.7%	*<0.001*
	s-RAGE (pg/ml)	590.2 ± 110.3	633.3 ± 221.6	40.9 ± 170.9	6.6 ± 27.4%	0.1656
	AGE/s-RAGE (µg/pg)	0.043 ± 0.017	0.018 ± 0.013	-0.024 ± 0.020	-53.1 ± 29.4%	*<0.001*
*P^‡^*, Pioglitazone *vs* Glimepiride						
	AGE	0.1291	0.1343	*0.0236*		
	s-RAGE	0.5239	0.4135	0.6043		
	AGE/s-RAGE	0.0912	0.2138	*0.0123*		

^†^ *P*, comparison 5-year vs basal, Student’s *t* test for paired data ^‡^ *P*, comparison between groups for basal, 5-year and Δ, Student’s *t* test for unpaired data.

Merging the data of the two groups (**Table 3**), the levels of AGE and AGE/s-RAGE ratio were both significantly reduced (basal compared to 5-year values: AGE 26.7 ± 12.2 µg/ml *vs* 9.0 ± 6.9 µg/ml, *P*<0.001; AGE/s-RAGE ratio 0.047 ± 0.022 µg/pg *vs* 0.016 ± 0.014 µg/pg, *P*<0.001). No significant difference was found for s-RAGE (583.5 ± 102.4 *vs* 614.3 ± 191.0 pg/ml, *P*=0.1086). The 5-year treatment with either drug confirmed an overall significant decrease of ΔAGE (Δ= -17.7 ± 12.8 µg/ml, *P*<0.001) and ΔAGE/s-RAGE ratio (Δ= -0.031 ± 0.022 µg/pg, *P*<0.001), corresponding to a decrease of the parameters by about 60%. The overall distribution of Δ values is shown in the dendrogram of **Supplementary Figure 1**. **Supplementary Figure 2** shows the distribution of the glycoxidation deltas values between females and males; no significant difference was found.

**Table 3:** AGE, s-RAGE and AGE/s-RAGE data considering patients altogether at both considered times.

Parameter	basal	5-year	Δ 5-year - basal	Δ % 5-year - basal	*P^†^*
AGE (µg/ml)	26.7 ± 12.2	9.0 ± 6.9	-17.7 ± 12.8	-61.2 ± 30.3%	*<0.001*
s-RAGE (pg/ml)	583.5 ± 102.4	614.3 ± 191.0	30.9 ± 159.9	5.6 ± 26.2%	0.1086
AGE/s-RAGE (µg/pg)	0.047 ± 0.022	0.016 ± 0.014	0.031 ± 0.022	-61.8 ± 29.3%	*<0.001*

^†^Differences between the two time points were evaluated with Student’s *t* test for paired data.

Again, considering all the patients, a possible correlation among differences in the available parameters at the end of the 5-year treatment was evaluated (**Supplementary Table 1**), conditioned on Δ Weight as confounding variable. The calculated 10-year ASNCVD risk did not correlate with any delta of AGE-RAGE parameters; as well, no significant correlation for other variables with deltas of AGE-RAGE parameters was detected (**Supplementary Table 1**).

## 4 Discussion

This study demonstrates a reduction in cardiovascular risk indicators AGE and AGE/s-RAGE ratio achieved by therapeutic intervention with both pioglitazone and glimepiride, in combination with metformin, independent of glycemic control. Patients on pioglitazone achieved a greater benefit, likely related to the characteristic effect of the drug on PARPγ receptors that activates antioxidant enzymes, including glutathione peroxidase, which contributes to the decrease in oxidative stress (29). In the present study, the s-RAGE parameter does not appear to be influenced by either pioglitazone or glimepiride treatment, glyco-metabolic control or inflammatory status, similar to previous studies (29–31) that indicated the independence of s-RAGE values from glycemia, glycaemic control, or insulin resistance, while reporting, unlike our observation, an increase in circulating levels of s-RAGE in patients taking pioglitazone. Adeshara et al. (32), in contrast, observed a reduction in circulating s-RAGE levels in metformin-treated patients; Nakamura et al. (33), in agreement with the present study, reported a reduction in plasma AGE levels with glimepiride, but only a reduction trend of s-RAGE. Therefore, the effect of oral hypoglycemic therapies on s-RAGE levels in the diabetic patient still appears controversial.

Several studies have shown that patients at higher cardiovascular risk (including subjects with T2DM) present a higher AGE/s-RAGE ratio than controls (22, 23, 25, 31, 34). More recently, a study confirmed that AGE and s-RAGE, as well as AGE/s-RAGE ratio are increased in T2DM, in comparison to healthy controls, also linked to the fact that c-RAGE fraction is increased (35); the same study concluded that circulating AGE and soluble RAGE isoforms can be predictors of major adverse cardiovascular events and all-cause mortality in subjects with T2DM, also with a positive association with age. In agreement with Sabbatinelli et al. (35) findings, our study shows a reduction in AGE/s-RAGE ratio, consistent with the absence of cardiovascular events over the 5 years of observation in all patients; however, the ASCVD risk, determined at baseline, did not correlate with any variation of the glyco-oxidation parameters. Also, no significant correlation was detected between the glyco-oxidation parameters and age of patients, nor with the duration of the disease, gender and other clinical parameters.

Elevated s-RAGE level is associated with the development of early and late renal disease both in the population not affected by diabetes (4, 36, 37), as well as in people with diabetes (29, 30). Other studies indicated that reduced s-RAGE levels in patients affected by diabetes correlate with the development of renal complications (34, 38). It remains to be determined whether increased s-RAGE is caused by decreased renal function (34), or whether s-RAGE levels are up-regulated to protect against the toxic effects of AGEs (39). To overcome the conflicting interpretations reported in the literature, the present study considered not only the s-RAGE parameter as a risk marker, but also the AGE/s-RAGE ratio, which was found to decline after 5 years of treatment, alongside preserved renal function, in the absence of correlation between the two parameters.

In the present study, despite a modest significant increase of body weight and BMI at the end of the 5-year period in the group treated with pioglitazone, no correlation was observed in the entire cohort between Δ BMI and the glyco-oxidation parameters of the AGE/RAGE axis. Indeed, several studies have been conducted to investigate the functioning of the AGE/RAGE axis in relation to body composition, weight and BMI, and the impact that weight, dietary and bariatric surgery interventions may have on cardiovascular risk reduction. Although there is still no conclusive evidence to support the cause-and-effect relationship between obesity and high AGE levels, a close association between AGEs and caloric intake has been reported (40), and on the other hand, chronic exposure to a diet high in AGEs promotes chronic inflammation and insulin resistance, all conditions known to favor obesity (41, 42). An association between increased RAGE expression and lipid accumulation in various cells and tissues has been reported (43), in particular in the adipose tissue of obese subjects (44); RAGE has also been shown to play a role in adipocyte hypertrophy and insulin resistance in animal models (45). Several studies have shown reduction of total s-RAGEs in individuals with obesity compared with normal-weight subjects (46); total s-RAGEs are negatively associated with BMI (47–52) and, in addition, overweight people with higher BMI have lower plasma levels of s-RAGE than normal-weight controls (52).

Studies that evaluated the impact of diet and bariatric surgery demonstrated, during follow-up of bariatric surgery, an increase in s-RAGE of 20% (47); in obese subjects undergoing dietary intervention over a 6-month period, serum levels of s-RAGE resulted significantly and inversely associated with BMI (53) and higher baseline levels of s-RAGE, prior to surgery, were predictors of improved parameters of T2DM (54), as well as of a greater weight loss (55). It is possible to conclude that, in the context of obesity, the AGE-RAGE axis is influenced by the increased presence of AGE, overexpression of RAGE, and reduced concentration of circulating s-RAGE, which is supposed to act as a scavenger. The present study supports the view that targeted regulation of s-RAGE might be a promising avenue for the treatment of obesity and its comorbidities (56), in line with the results obtained by Parikh et al. (55) which support the hypothesis that s-RAGE receptors are potential new markers for identifying obese patients who might benefit most from weight management interventions. According to Miranda et al. (50) weight loss preserves the pool of circulating s-RAGE, confirming the protective effects of weight loss, and strengthening the hypothesis (48) that increased levels of s-RAGE have protective value against lipid accumulation because they prevent overexpression of RAGE.

A key question remains whether interventions to modulate AGE-RAGE axis might provide a protection against atherosclerosis progression. However, a major strength of this study consists in its structure as a longitudinal observation that permitted a five-year duration, in order to investigate specific glyco-oxidation parameters which are usually evaluated by cross-sectional studies only.

Our data exclude a correlation of the calculated 10-year ASNCVD risk with ΔAGE-RAGE parameters; interestingly, while in the literature a correlation is highlighted between AGE-RAGE axis and several cardiovascular risk factors when considered separately (57), this correlation has not been found in view of a cardiovascular risk estimation model bringing together multiple risk factors. This finding could be explained on the one hand by the very high calculated cardiovascular risk and on the other by the presence in the score estimation of classic risk factors less correlated to the AGE-RAGE axis. The relevance of the measured parameters as indicators of cardiovascular and renal risk factors would possibly be considered in future development of the investigation, with a longer follow-up, where specific target organ impairment could be evaluated and correlated. Significant gender-related differences as well were not observed in both groups of the present study, in agreement with a previous published report (35). From literature data, not even the RAGE genetic variants were shown to be associated with the incidence of cardiovascular disease in subjects affected by diabetes (58).

The present study has limitations, in particular linked to the small sample size and the follow-up limited to 5 years, all factors that depends on the fact that the research was derived from a main project multicenter clinical trial (TOSCA.IT) with strict limitations of the protocol. The same protocol did not involve a control group. The data here observed, although significant, should be in the future straightened with longer follow-up under the two treatment regimens, including a placebo group, regarding the values of AGE, s-RAGE and AGE/s-RAGE ratio, also in relation to the possible occurrence of related cardiovascular complications.

In conclusion, the present study evaluated the AGE/s-RAGE ratio in a cohort of patients with T2DM on different oral hypoglycaemic therapies: metformin in combination with pioglitazone or glimepiride. Taking into consideration both the entire cohort of patients and the groups separately, the AGE/s-RAGE ratio was significantly reduced, a result that, in agreement with what is reported in the literature (23–25), can be interpreted as an index of cardiovascular risk reduction. In the cohort of patients investigated there were also no significant changes in parameters assessing clinical, metabolic, renal function, pro-inflammatory status and glycaemic control. Treatment with pioglitazone seems to offer greater benefit on the AGE/s-RAGE ratio as disease progression indicator. Pioglitazone should be considered not only for its known favorable effects on established CV risk factors (59), but also because of its long-term action on the AGE/sRAGE axis, as shown in the present investigation.

The indications arising from the present research suggest future directions of investigation that include a careful re-evaluation of the benefits of antidiabetic therapy with classical drugs of known and proven hypoglycaemic properties. Possible pleiotropic actions, although still hidden, may reveal their usefulness in counteracting the onset of diabetes-related complications, especially with the aim of containing the cardiovascular risk associated with the disease. The usefulness of the AGE/s-RAGE ratio for disease monitoring might suggest to examine its use as an additional parameter to be added to the current panel of biohumoral markers of diabetes control.

## Data availability statement

The raw data supporting the conclusions of this article will be made available by the authors, without undue reservation.

## Ethics statement

The study was conducted in accordance with the Declaration of Helsinki and the EU guidelines on Good Clinical Practice for conducting clinical trials of medicines; the study was approved by the Ethics Review Committee/Institutional Review Board of the Coordinating Center and by the Ethics Committees of each individual participating center of TOSCA.IT (Thiazolidinediones Or Sulphonylureas and Cardiovascular Accidents. Intervention Trial) Trial, registered at ClinicalTrials.gov as NCT00700856 (https://www.clinicaltrials.gov/ct2/show/NCT00700856). The patients/participants provided their written informed consent to participate in this study.

## Author contributions

GS, AL and ER contributed to the conception and design of the research; SB and NCC conducted investigation and data collection; CC performed analytical determinations; ER performed statistical data analysis; GS and ER wrote sections of the manuscript. All authors contributed to the article and approved the submitted version.
