# Supplementary material for: Anti-diabetic combination therapy with pioglitazone or glimepiride added to metformin on the AGE-RAGE axis: a randomized prospective study
Source: Front Endocrinol (Lausanne). 2023 Aug 11;14:1163554. doi: 10.3389/fendo.2023.1163554 (PMC10453795; doi:10.3389/fendo.2023.1163554)
Supplement: Supplementary file 1 [file DataSheet_1.pdf]

frontiers

Supplementary Material

Anti-diabetic combination therapy with pioglitazone or glimepiride added to metformin on the AGE-RAGE axis: a randomized prospective study

Eugenio Ragazzi[1]*, Silvia Burlina[2], Chiara Cosma[2], Nino Cristiano Chilelli[2], Annunziata Lapolla[2] and Giovanni Sartore[2]

[1]Department of Pharmaceutical and Pharmacological Sciences, University of Padova, Padova, Italy

[2]Department of Medicine – DIMED, University of Padova, Padova, Italy

*** Correspondence:**
Corresponding Author: eugenio.ragazzi@unipd.it

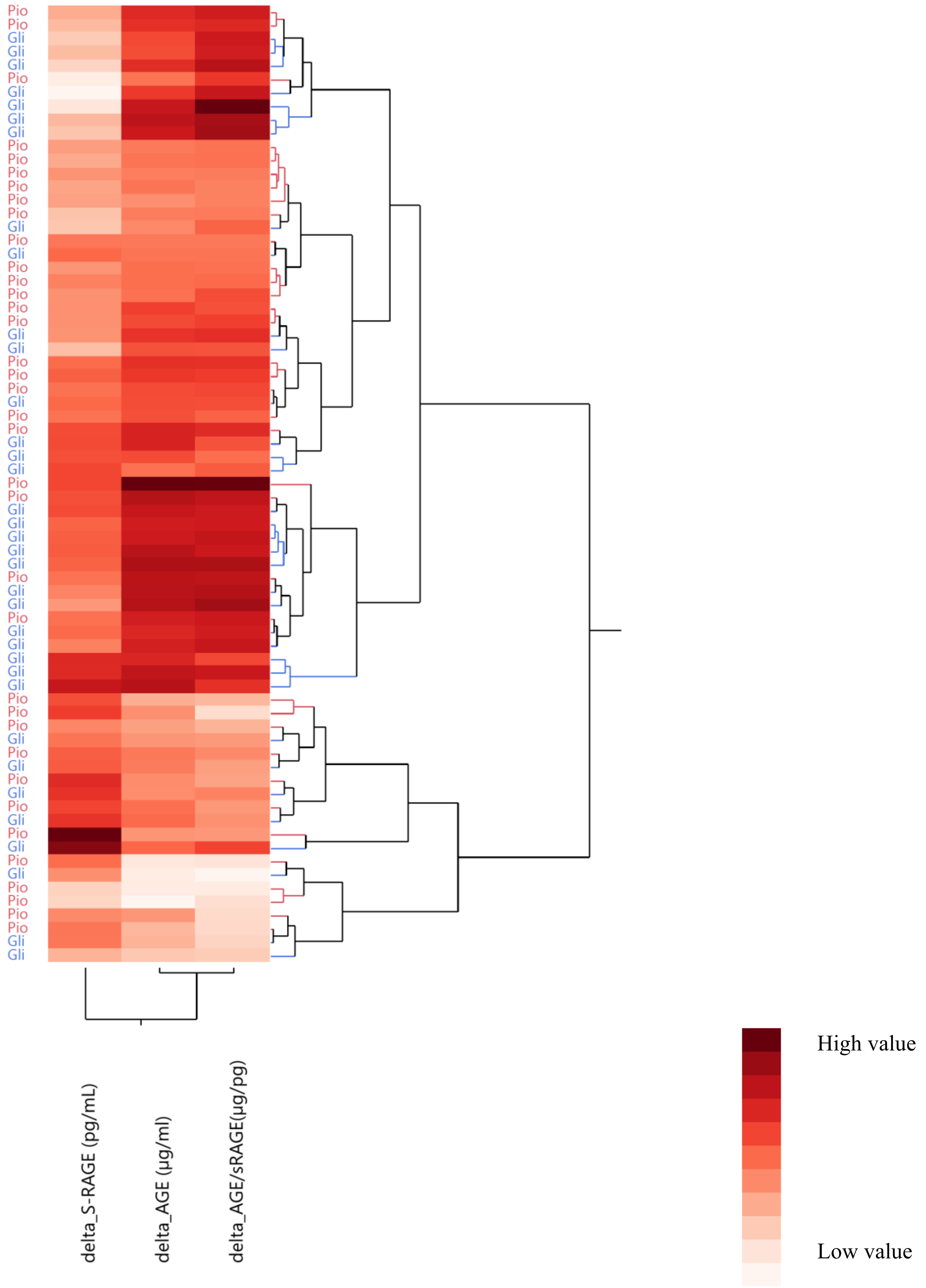

Supplementary Figure 1. Dendrogram and related heatmap obtained with cluster analysis, representing the distribution of Δ glyco-oxidation parameters among patients of the two treatment groups (Pio: pioglitazone; Gli: glimepiride). Cluster analysis was performed according to Ward's agglomerative hierarchical procedure, with values standardized by each variable. Heatmap color intensity is proportional to the parameter's value, as in the reported scale.

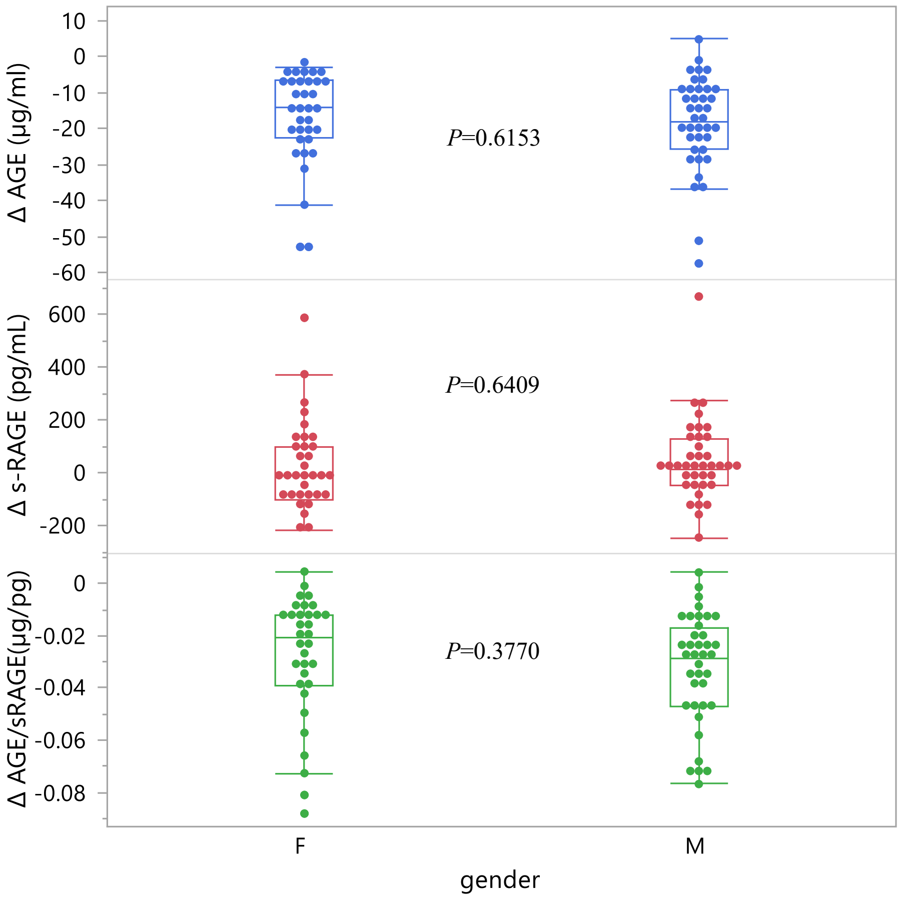

Supplementary Figure 2. Distribution of Δ glyco-oxidation parameters in the overall cohort of patients according to gender. The edges of the boxes indicate the 25th and 75th quantiles, including the middle 50 percent of the data; whiskers indicate the furthest points within 1.5 x IQR from the box. IQR is the interquartile range, defined as the difference between the 75th and 25th percentiles. Statistical significance is indicated for each parameter.

Supplementary Table 1. Paired correlations (linear model) for Δ of the various parameters, considering all patients, and conditioned on ΔWeight as confounding variable.

Variable	vs Variable	Correlation coefficient *r*	*P*
Age	Δ_S-RAGE	−0.210	0.080
Age	Δ_AGE	−0.036	0.767
Age	Δ_AGE/sRAGE	−0.016	0.894
Diabetes duration	Δ_S-RAGE	0.013	0.916
Diabetes duration	Δ_AGE	0.177	0.137
Diabetes duration	Δ_AGE/sRAGE	0.198	0.100
Δ BMI	Δ_S-RAGE	−0.104	0.392
Δ BMI	Δ_AGE	0.171	0.150
Δ BMI	Δ_AGE/sRAGE	0.187	0.121
Δ SBP	Δ_S-RAGE	−0.213	0.077
Δ SBP	Δ_AGE	−0.057	0.633
Δ SBP	Δ_AGE/sRAGE	−0.043	0.725
Δ DBP	Δ_S-RAGE	−0.179	0.139
Δ DBP	Δ_AGE	0.067	0.573
Δ DBP	Δ_AGE/sRAGE	0.145	0.231
Δ Waist	Δ_S-RAGE	0.034	0.780
Δ Waist	Δ_AGE	0.084	0.481
Δ Waist	Δ_AGE/sRAGE	0.209	0.083
Δ eGFR	Δ_S-RAGE	0.079	0.515
Δ eGFR	Δ_AGE	0.080	0.507
Δ eGFR	Δ_AGE/sRAGE	0.076	0.534
Δ Serum creatinine	Δ_S-RAGE	−0.086	0.479
Δ Serum creatinine	Δ_AGE	−0.023	0.849
Δ Serum creatinine	Δ_AGE/sRAGE	−0.042	0.728
Δ HbA1c	Δ_S-RAGE	0.121	0.318
Δ HbA1c	Δ_AGE	−0.010	0.935
Δ HbA1c	Δ_AGE/sRAGE	0.004	0.976
Δ CRP	Δ_S-RAGE	0.105	0.385
Δ CRP	Δ_AGE	−0.022	0.857
Δ CRP	Δ_AGE/sRAGE	0.0008	0.995
10y-ASCVD risk	Δ_S-RAGE	0.022	0.856
10y-ASCVD risk	Δ_AGE	−0.036	0.762
10y-ASCVD risk	Δ_AGE/sRAGE	−0.047	0.697

Note. Conditioned on variables: Δ Weight
